# Sex Differences in the Effect of Type 2 Diabetes on Major Cardiovascular Diseases: Results from a Population-Based Study in Italy

**DOI:** 10.1155/2017/6039356

**Published:** 2017-02-20

**Authors:** Paola Ballotari, Francesco Venturelli, Marina Greci, Paolo Giorgi Rossi, Valeria Manicardi

**Affiliations:** ^1^Interinstitutional Epidemiology Unit, Local Health Authority of Reggio Emilia, Via Amendola 2, 42122 Reggio Emilia, Italy; ^2^Arcispedale Santa Maria Nuova-IRCCS, Reggio Emilia, Viale Umberto I 50, 42123 Reggio Emilia, Italy; ^3^Department of Biomedical, Metabolic and Neural Sciences, University of Modena and Reggio Emilia, Via Campi 287, 41126 Modena, Italy; ^4^Primary Care Department, Local Health Authority of Reggio Emilia, Via Amendola 2, 42122 Reggio Emilia, Italy; ^5^Internal Medicine Department, Montecchio Hospital, Local Health Authority of Reggio Emilia, Via Barilla 16, 42027 Montecchio, Italy

## Abstract

The aim of the study is to assess sex difference in association between type 2 diabetes and incidence of major cardiovascular events, that is, myocardial infarction, stroke, and heart failure, using information retrieved by diabetes register. The inhabitants of Reggio Emilia (Italy) aged 30–84 were followed during 2012–2014. Incidence rate ratios and 95% confidence intervals were calculated using multivariate Poisson model. The age- and sex-specific event rates were graphed. Subjects with type 2 diabetes had an excess risk compared to their counterparts without diabetes for all the three major cardiovascular events. The excess risk is similar in women and men for stroke (1.8 times) and heart failure (2.7 times), while for myocardial infarction, the excess risk in women is greater than the one observed in men (IRR 2.58, 95% CI 2.22–3.00 and IRR 1.78, 95% CI 1.60–2.00, resp.; *P* of interaction < *0.0001*). Women had always a lesser risk than men, but in case of myocardial infarction, the women with type 2 diabetes lost part of advantage gained by women free of diabetes (IRR 0.61, 95% CI 0.53–0.72 and IRR 0.36, 95% CI 0.33–0.39, resp.). In women with type 2 diabetes, the risk of major cardiovascular events is anticipated by 20–30 years, while in men it is by 15–20.

## 1. Introduction

In the list of the top ten killers created by the Global Burden of Disease study, ischemic heart disease and stroke contended for first place, causing 13% and 12% of the total deaths in 2012, respectively. Moreover, the WHO estimated that ischemic heart disease contributed to a third of the 96.4% increase in the prevalence of heart failure (HF) from 1990 to 2013 worldwide [1].

Accordingly, the European Society of Cardiology (ESC) identifies cardiovascular diseases (CVDs) as the leading cause of death in Europe. In a recent issue, the ESC estimated that, despite recent decreases in mortality rate in many countries, close to half deaths in Europe in 2014 are attributable to CVDs, with a higher proportion in women (51%) than in men (42%) [2].

The WHO also identified diabetes as the fifth and ninth cause of deaths in women and men, respectively [1]. Indeed, diabetes is a major public health issue with an increasing prevalence globally, affecting at least 8% of the adult population worldwide [3].

Cardiovascular diseases, including coronary heart disease (CHD), stroke, and heart failure, are predominant causes of morbidity and mortality among people with diabetes [4].

Conversely, diabetes increases the prevalence of most of the main risk factors for CVDs, leading to an increased risk of related morbidity and mortality [5–12]. However, accruing evidence highlights that women and men experience the disease differently [13, 14]. Actually, women lose their relative protection from CVDs, and postmenopausal diabetic females, compared to the general population, presented a stronger increase of cardiometabolic risk than diabetic males, but the reasons are not entirely clear [15–18]. The explanations are likely to be multifactorial, with contributions from differences in inherent physiological factors and in the management and treatment of diabetes, to the detriment of women [19, 20].

The data collected by the Italian Society of Diabetologists (AMD) on quality of diabetes care and gender difference in access to effective care in Italy, show, in a large population of type 2 diabetes, that gender disparities in control of the disease are less pronounced in Italy than in other countries, but they still exist, despite equity of access to specialist care and the same pharmacological treatment of women and men [21].

A specific negative interaction between being a woman and having diabetes in CVDs risk was supported by most of the studies [22–25] but questioned by some other authors [18, 26]. The inconsistency of evidence could be partly due to several limitations such as failure to distinguishing between types of diabetes or selection bias in observational studies or extremely selected populations in trial participants [27, 28]. Large register-based studies which identify the different types of diabetes are required to examine differences between sex in CVD occurrence and mortality in the general population.

In the Reggio Emilia province, the Diabetes Register (REDR) was set up since 2009 and is able to ascertain cases and to distinguish the type of diabetes [29].

The present study aims to assess sex differences in the association between type 2 diabetes (T2D) and incidence of major cardiovascular diseases (CVDs), that is, myocardial infarction, stroke, and heart failure, using the REDR information.

## 2. Materials and Methods

### 2.1. Setting and Study Population

This is a cohort study where all residents in the Reggio Emilia province, Italy, as of December 31st, 2011 (approx. 0.5 million inhabitants), aged 30–84, were followed during 2012–2014 period. The follow-up lasted until the date of first CVD event, all-causes death, emigration, or end of follow-up (as of December 31st, 2014), whichever occurred first.

The residence and vital status information was retrieved from the civil register, as well as T2D status from the Reggio Emilia Diabetes Register (REDR).

The REDR is a validated database created by the deterministic linkage of six routinely collected data sources through a definite algorithm able to ascertain cases and to distinguish type of diabetes and model of care [29]. Data have been included since 2009, and the REDR is updated annually. The date of inclusion in the register is the date when a person first meets one of the following inclusion criteria: (1) disease-specific exemption database: exemption from copayment due to diabetes; (2) hospital discharge database: hospitalization with diabetes diagnosis in whichever position by ICD-9 (International Classification of Diseases Clinical Modification, 9th Edition) codes 250.xx, 357.2x, 362.0x, 366.41, and 648.0x, excluding MDC14; (3) biochemistry laboratory database: one glycated haemoglobin (HbA1c) test ≥ 6.5% (48 mmol/mol); (4) drug prescription databases: redeemed prescription at least twice for antidiabetic drugs in case of pharmacy distribution, only one in case of direct distribution; (5) diabetes outpatient clinics database: diagnosis by a diabetologist; and (6) Reggio Emilia mortality register: cause of death by ICD-10 (International Classification of Diseases, 10th Edition) codes E10–E14. Women with gestational diabetes or women receiving treatment for polycystic ovarian syndrome were excluded.

The type of diabetes, suggested by the presence of defined criteria as described elsewhere [29], was confirmed by a diagnosis provided by a diabetologist or another physician.

Subjects included in the register with type 1 diabetes or secondary diabetes (i.e., drug-induced diabetes, diseases of exocrine pancreas, etc.) or with undefined diabetes were excluded from the analysis.

### 2.2. Outcomes and Other Variables

The outcomes were the first event between hospitalization and death for stroke, myocardial infarction, and heart failure. Fatal events were considered cases in which only the death certificate was present or cases with hospitalization followed by a death certificate for the same cause during the study period. The outcomes were defined according to the International Classification of Disease, 9th Revision (ICD-9) in case of nonfatal event and 10th Revision (ICD-10) in case of fatal event: (1) nonfatal stroke (ICD-9 codes 430–434) or death from cerebrovascular disease (ICD-10 codes I60–I63); (2) nonfatal myocardial infarction (ICD-9 codes 410‐411) or deaths from myocardial infarction (ICD-10 codes I21, I22, I24.8, I24.9); and (3) heart failure requiring hospitalization (ICD-9 code 428) or causing death (ICD-10 code I50).

Data were retrieved from hospital discharge database in case of nonfatal events, using primary diagnosis and excluding day hospital, and from Reggio Emilia mortality register for deaths. Each participant could contribute only once to incidence of each event, that is, with first event detected in the study period, but the same subject could contribute for more types of outcome.

Individual demographic information included age, sex, and foreign status (i.e., non-Italian citizenship).

### 2.3. Statistical Methods

The characteristics of the study population are presented as median and proportions and stratified by sex and T2D status. The event numbers and person-time at risk were calculated for each major CVD examined.

Age-adjusted event rates (AAER) per 10,000 with 95% confidence intervals (95% CI) by sex and T2D status for each type of event were estimated using the Italian population as of December 31st, 2011, as a reference for standardization [30]. Incidence rate ratios (IRR) and 95% confidence intervals (95% CI) for the risk of each CDV in people with T2D versus the population without diabetes were calculated using multivariate Poisson regression models and stratifying by sex. The effect modification of sex on the association between T2D and CVDs was tested using the Wald test. Finally, the age-specific event rates stratified by sex for individuals with and without diabetes were graphed.

The analyses were performed using the STATA statistical package Version 13.0.

## 3. Results

On December 31st, 2011, the REDR included 14,531 and 12,424 prevalent men and women with T2D, respectively. The overall prevalence is 5.7% and 4.7%, respectively. Restricting to people aged 30–84, 13,714 men and 10,634 women (44% out of the total) with T2D were included in our study (Table 1). The median age of the diabetic population is about 20 years higher than the people without diabetes.

During the 3-year follow-up, the number of fatal events was higher for myocardial infarction than for other CVDs (Table 2) (9.6% and 9.1% for men and women, resp., with no difference between the two sexes). Among men without T2D, myocardial infarction was the most frequent CVD, closely followed by stroke and remotely by heart failure, while among women without T2D, the stroke was the most frequent CVD, followed by myocardial infarction and heart failure, both halved with respect to the former.

Performing the analysis without sex stratifications, the stroke and myocardial infarction risks in people with T2D were almost twofold higher than in those without diabetes, while heart failure risk was almost three times higher (IRR 1.84, 95% CI 1.70–1.98; IRR 2.00, 95% CI 1.83–2.18; IRR 2.70, 95% CI 2.47–2.94, resp., data not reported in the tables).

Women with T2D had 1.8 times the probability of a stroke (Table 2) than the women without diabetes (95% CI 1.61–2.04), 2.6 times the probability of having a myocardial infarction and heart failure (95% CI 2.22–3.00 and 2.27–2.97, resp.). Men with T2D have similar excess risk for stroke and heart failure, but the excess risk for myocardial infarction is lower than the one observed in women (IRR 1.78, 95% CI 1.60–2.00).

The relative risk for myocardial infarction associated with T2D was significantly greater in women than in men (*P* < 0.0001), while there is no evidence of sex difference for the stroke and heart failure (*P* = 0.9151 and *P* = 0.9289, resp.). Women free of diabetes had lesser risk than men free of diabetes to experience whichever CVD events (Table 3). The reduction is 30% in case of stroke and heart failure and 60% in case of myocardial infarction. Women with type 2 diabetes had also lesser risk than men with type 2 diabetes to experience whichever CVD events, but in case of stroke and heart failure, the reduction percentages were similar to those found comparing sex free of diabetes; meanwhile, in case of myocardial infarction, the reduction is only 30% (instead of 60%).

The foreigners seemed to have similar risk of stroke (in people with T2D IRR 0.93, 95% CI 0.62–1.40; in people without T2D IRR 0.90, 95% CI 0.72–1.12) and heart failure (in people with T2D IRR 0.76, 95% CI 0.45–1.28; in people without T2D IRR 0.98, 95% CI 0.69–1.40), while in case of myocardial infarction, a protective effect was found in the population without T2D, but not in those with T2D (in people with T2D IRR 1.03, 95% CI 0.71–1.48; in people without T2D IRR 0.68, 95% CI 0.54–0.86). The same pattern was observed in men and women.

**Figure 1:**
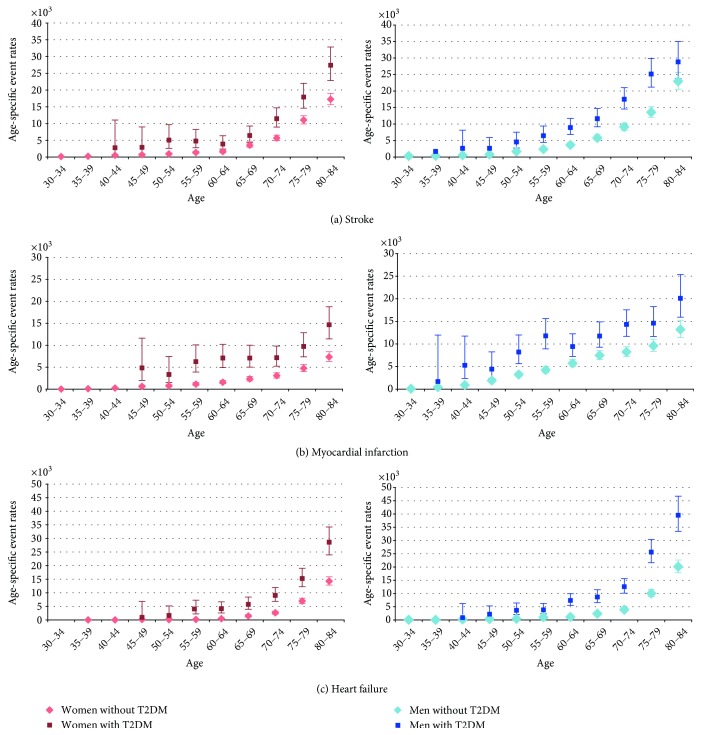
Age-specific event rates by sex, type 2 diabetes status, and type of event.

In the women without diabetes, the risk is virtually zero until 54 yrs for myocardial infarction, until 60 for stroke and until 70 for heart failure (Figure 1; Annex 1 in Supplementary Material available online at https://doi.org/10.1155/2017/6039356), while for the men without diabetes, the risk is appreciable from the age of 45 for myocardial infarction, 50 for stroke, and 65 for heart failure. In general, the risk of CVD events in men and women with T2D starts to be noticeable at an earlier age than in the population without diabetes. The anticipation is longer in women than in men for myocardial infarction and stroke. The resulting excess risk for people with T2D is therefore higher in younger ages for all the outcomes in both sexes and, particularly, for myocardial infarction in women.

## 4. Discussion

In our study, people with T2D experienced an excess risk for all the investigated CVDs, that is, stroke, myocardial infarction, and heart failure. These results are consistent with many studies [13, 14] and reflect those observed for mortality in a previous analysis applied to the same population, where we found an excess risk for cardiovascular diseases in population with diabetes of both sexes, stronger in women and in particular for myocardial infarction [31].

Although we observed nondiabetic women having fewer CVD events than nondiabetic men of the same age, this advantage appears to be partially lost for myocardial infarction in the context of T2D, but not for the other two CVDs. Our results were consistent with those of a recent Italian study that showed a diabetes-related excess risk greater in women than in men for myocardial infarction but not for heart failure and stroke [32].

In a Finnish cohort study [13], the presence of diabetes reduced the so-called female advantage for CVD risk; indeed, mortality from CHD was three times higher in women compared with men with diabetes. Similar findings were observed in the NHANES cohort [33] and in the study based on UK General Practice data [34], the latter investigating subsequent myocardial infarction. The stronger impact of diabetes as a major risk factor for CVD events in women than men, 4.3-fold risk compared to 2.7, is described also in the INTERHEART, an international case-control study including 15,152 cases and 14,820 controls from 52 countries [35]. On the other hand, a systematic review showed that the risk of incident CHD was three times higher in women with diabetes than in women without diabetes, while the risk in men doubled [36]. A similar difference in the excess of risk, 50% more, was found in a previous systematic review [15].

We observed a risk of heart failure almost 3 times higher in the presence of T2D, similar to results found in Oregon [10] and in Iceland [37], but in our study, the overall excess risk was similar in women and men (*P* = 0.9245). In the Framingham Heart Study [25], the heart failure risk was 2 times higher in men and 5 times higher in women with diabetes compared with the general population.

Also, in case of stroke, we found an excess risk for T2D but no sex difference in the excess (*P* = 0.9197). The results of individual studies on the sex differences on the risk of stroke associated with diabetes have been inconsistent, with some studies showing women with diabetes had higher [13, 25, 38, 39], similar [40], or lower risk [41, 42] compared to men with diabetes. In a recent comprehensive systematic review and meta-analysis using data from 64 cohorts [20], the pooled analysis shows a significantly higher relative effect of diabetes on stroke risk in women compared to men, even adjusting for other major cardiovascular risk factors. As suggested by other authors [32], the different prevalence of medical condition underlying the physiopathology of stroke (i.e., arterial hypertension and atrial fibrillation) between sexes [43] may reduce the difference in the gender-related diabetes risk for stroke and myocardial infarction.

This greater excess coronary risk may be explained by more adverse cardiovascular risk profiles among women with diabetes, combined with possible disparities in treatment and quality of care that favor men. A recent review explored biological and environmental factors that play a role in the reduction of the protective effect of female sex from CVD in diabetic women [44]. The authors pointed out the interaction between insulin and the estrogen signaling and the effect of hyperglycemia on the expression and activity of estrogen receptors as mechanisms underlying the diabetes-related impairment of endothelium in diabetic women. The resulting proinflammatory environment accelerates the atherosclerotic process that leads to coronary arteries disease, particularly in women.

In two large population studies carried out in Italy [21, 45], subjects with T2D—included the majority of the population enrolled our study—were investigated to ascertain whether gender differences in quality of care of diabetes exist in Italy [21]. The authors found that the likelihood to reach specific clinical outcomes is systematically unfavorable for women as compared with men, although the disparities are less pronounced than in other countries.

Many international studies documented a systematic undertreatment of cardiovascular risk factors in diabetic women: the results of the DIANA study [46] indicated that men with diabetes were significantly more likely to receive any treatment for the major CVD risk factors, including oral hypoglycemic agents, ACE inhibitors, and calcium channel blockers for CHD, than women. Evidence has emerged demonstrating a potential sex disparity in the intensity of cardiovascular risk reduction, whereby worse glycated hemoglobin control, lower frequency of lipid-lowering therapy, lower aspirin use, and lower blood pressure control were noted in women [47].

In contrast with these international data [48], women with diabetes in Italy are not undertreated with medications for cardiovascular risk factors. Also in the study of Rossi et al. [21], the proportion of diabetic women treated with insulin, statins, or antihypertensive agents was equal to or even higher than that of men. The MIND-IT (Multifactorial Intervention in Type 2 Diabetes in Italy)—a cross-sectional study that enrolled over 2,000 type 2 diabetic patients without previous described CVD events—investigated the degree of control of CVD risk factors [49]: diabetic women showed a worse CVD risk profile, with a less percentage of diabetic women reaching the recommended metabolic targets compared to men, regardless of use of medications for CVD risk factors control. In the light of reported evidence, we suggest that both the diverse physiopathology and the systematic use of ACE inhibitors or B-blockers and statins in Italian diabetic patients, without gender differences, might explain the lack of differences in stroke and heart failure risk between men and women.

The age curves of risk show that in the population with T2D the incidence is brought forward by 20–30 years in women and by 15–20 years in men for stroke and myocardial infarction, while for the heart failure, a marked anticipation of the onset can be observed for both sexes (Figure 1; Annex 1 in Supplementary Material). Consequently, the incidence of stroke and myocardial infarction in young ages is several times higher than the one in the population without diabetes, while in people over 70, it is only 2- or 1.5-fold. This is particularly true for women, because the incidence of stroke and myocardial infarction in the population without diabetes remains virtually null before the age of 60. The results are consistent with a previous study [13] in Finland.

### 4.1. Strengths and Limitation

The population-based cohort study approach increases the external validity, and the use of diabetes register reduces the ascertainment bias. Indeed, the study excludes cases of type 1 diabetes (732), secondary diabetes (137), or undefined diabetes (901), maximizing the accuracy in the definition of the two subcohorts (i.e., with and without T2D).

Although the AHA statement notes possible sex differences in cardiovascular outcomes within racial/ethnic groups, our study did not have sufficient power to analyze differences by geographical origin, although we use this covariate in multivariate model. In addition, we could not control for other cardiovascular risk factors in the analysis, because unlike observational studies of patients from clinical databases, there is only limited clinical information on the general population; hence, it is not possible to provide a detailed clinical characterization of the general population. We foresee a second study to investigate the determinants of CVD events only in population with T2D using different clinical information as well as prescribed drug and information on other areas of care such as tests and lifestyle factors and diabetes duration.

## 5. Conclusions

Women with T2D have earlier risk of major cardiovascular events by 20–30 years and males by 15–20 compared to the people without diabetes. The overall incidence of myocardial infarction, stroke, and heart failure is about twofold in T2D compared to the people without diabetes. The diabetes-related excess risk of myocardial infarction is much higher for women than for men, while for the other CVDs, people with T2D of the two sexes have a similar excess risk. In light of finding possible mechanisms to explain the partial loss of advantage for myocardial infarction and not for the other two types of events, we foresee a second study to investigate the determinants of CVD events only in population with T2D using information about prescribed drug and on other areas of care such as tests and lifestyle factors and diabetes duration.

## Supplementary Material

Annex1: N of event and five-yr-incidence rate ratios (IRR) with 95% Confidence Intervals (95% CI) by type of event, sex and age class.

## Figures and Tables

**Table 1:** Baseline characteristics of the study cohorts, age 30–84, Reggio Emilia—Italy, as of December 31st, 2011.

	Men		Women	
	Without T2DM	With T2DM	Without T2DM	With T2DM
Inhabitants				
Total, *n*	161,045	13,714	170,798	10,634
Foreigners, *n* (%)	17,444 (10.8)	907 (6.6)	19,336 (11.3)	846 (8.0)
Age (years): median (IQR)	48 (39–61)	67 (58–74)	50 (40–64)	70 (61–77)

T2D = type 2 diabetes.

**Table 2:** *N* of events, person-years, and age-adjusted event rates (AAER) per 10,000 person-years and incidence rate ratios (IRR) with 95% confidence intervals (95% CI) by sex and type of event.

Events	Men							Women						
	Without T2D			With T2D				Without T2D			With T2D			
	*N* of events (fatal)	Person-years	AAER	*N* of events (fatal)	Person-years	AAER	IRR (95% CI)	*N* of events (fatal)	Person-years	AAER	*N* of events (fatal)	Person-years	AAER	IRR (95% CI)
Stroke	1454 (95)	475,145.4	37.28	517 (36)	38,600.9	74.70	1.86 (1.68–2.06)	1301 (115)	506,105.0	30.10	341 (22)	30,187.2	61.73	1.81 (1.61–2.04)
Myocardial infarction	1590 (217)	474,943.0	39.04	459 (60)	38,716.2	78.02	1.78 (1.60–2.00)	713 (128)	506,799.3	16.13	241 (37)	30,328.7	47.58	2.58 (2.22–3.00)
Heart failure	816 (14)	475,993.8	21.47	481 (10)	38,702.4	63.71	2.78 (2.48–3.12)	718 (17)	506,803.8	17.10	306 (10)	30,262.9	48.83	2.59 (2.27–2.97)

Age-adjusted event rates were calculated using Italian population at December 31st, 2011, stratified by sex.

IRR = calculated using Poisson model, adjusted for age and foreign status. People without type 2 diabetes were used as reference.

**Table 3:** Incidence rate ratios (IRR) with 95% confidence intervals (95% CI) by type 2 diabetes status and type of event, women versus men.

Women versus men	Without T2DM		With T2DM	
	IRR	95% CI	IRR	95% CI
Stroke	0.68	0.63–0.74	0.72	0.62–0.82
Myocardial infarction	0.36	0.33–0.39	0.61	0.53–0.72
Heart failure	0.63	0.57–0.69	0.66	0.58–0.77

IRR = calculated using Poisson model, adjusted for age and foreign status. Men were used as reference.
