# Supplementary material for: Sex Differences in the Effect of Type 2 Diabetes on Major Cardiovascular Diseases: Results from a Population-Based Study in Italy
Source: Int J Endocrinol. 2017 Feb 20;2017:6039356. doi: 10.1155/2017/6039356 (PMC5338069; doi:10.1155/2017/6039356)
Supplement: Supplementary file 1 — Annex1: N of event and five-yr-incidence rate ratios (IRR) with 95% Confidence Intervals (95% CI) by type of event, sex and age class. [file 6039356.f1.pdf]

Annex1: N of event and five-yr-incidence rate ratios (IRR) with 95% Confidence Intervals (95% CI) by type of event, sex and age class.

	**Stroke**						**Myocardial infarction**						**Heart failure**					
	**Men**			**Women**			**Men**			**Women**			**Men**			**Women**		
**Age class**	**No T2D**	**T2D**	**IRR 95%CI**	**No T2D**	**T2D**	**IRR 95%CI**	**No T2D**	**T2D**	**IRR 95%CI**	**No T2D**	**T2D**	**IRR 95%CI**	**No T2D**	**T2D**	**IRR 95%CI**	**No T2D**	**T2D**	**IRR 95%CI**
30-34	16	0	-	7	0	-	5	0	-	1	0	-	5	0	-	0	0	-
35-39	24	1	4.64<br>0.61-35.01	15	0	-	30	1	4.35<br>0.59-32.31	7	0	-	4	0	-	2	0	-
40-44	36	3	4.95<br>1.50-16.38	34	2	4.18<br>0.98-17.9	62	6	5.72<br>2.44-13.40	16	0	-	16	1	3.53<br>0.46-27.28	2	0	-
45-49	48	6	3.42<br>1.45-8.07	42	3	4.34<br>1.34-14.11	118	10	2.22<br>1.16-4.25	39	5	6.67<br>2.61-17.09	22	5	5.92<br>2.21-15.82	10	1	4.69<br>0.59-37.31
50-54	83	15	2.68<br>1.54-4.67	49	9	5.37<br>2.63-10.99	160	27	2.41<br>1.60-3.63	39	6	4.54<br>1.91-10.77	22	12	7.73<br>3.81-15.72	6	3	14.76<br>3.67-59.30
55-59	96	27	2.64<br>1.72-4.05	60	13	3.55<br>1.94-6.50	172	49	2.75<br>2.00-3.77	51	17	5.77<br>3.32-10.01	42	16	3.61<br>2.02-6.43	8	11	21.20<br>8.50-53.02
60-64	138	53	2.41<br>1.75-3.31	70	16	2.33<br>1.35-4.01	215	56	1.66<br>1.24-2.23	68	29	4.43<br>2.87-6.86	43	44	6.51<br>4.28-9.92	20	17	8.93<br>4.67-17.08
65-69	170	68	1.99<br>1.50-2.63	120	29	1.78<br>1.18-2.67	220	69	1.57<br>1.20-2.06	80	32	3.06<br>2.03-4.62	70	51	3.60<br>2.51-5.17	50	26	3.88<br>2.41-6.23
70-74	250	113	1.91<br>1.53-2.39	191	62	2.01<br>1.51-2.69	226	93	1.74<br>1.36-2.21	104	39	2.32<br>1.60-3.35	107	82	3.23<br>2.42-4.31	89	49	3.35<br>2.36-4.75
75-79	278	130	1.86<br>1.51-2.29	307	91	1.62<br>1.28-2.05	198	77	1.52<br>1.17-1.98	133	50	2.04<br>1.48-2.83	207	133	2.56<br>2.06-3.18	194	78	2.18<br>1.68-2.84
80-84	315	101	1.26<br>1.00-1.57	406	116	1.60<br>1.30-1.96	184	71	1.53<br>1.16-2.00	175	63	2.00<br>1.50-2.66	278	137	1.98<br>1.62-2.43	337	121	2.00<br>1.63-2.47
Total	*1454*	*517*	*1.86*<br>*1.68-2.06*	*1301*	*341*	*1.81*<br>*1.61-2.04*	*1590*	*459*	*1.78*<br>*1.60-2.00*	*713*	*241*	*2.58*<br>*2.22-3.00*	*816*	*481*	*2.78*<br>*2.48-3.12*	*718*	*306*	*2.59*<br>*2.27-2.97*

T2D = type 2 diabetes; IRR = calculated using Poisson model, adjusted for age and foreign status. People without type 2 diabetes was used as reference
